# Generating evidence on a risk-based monitoring approach in the academic setting – lessons learned

**DOI:** 10.1186/s12874-017-0308-6

**Published:** 2017-02-14

**Authors:** Belinda von Niederhäusern, Annette Orleth, Sabine Schädelin, Nawal Rawi, Martin Velkopolszky, Claudia Becherer, Pascal Benkert, Priya Satalkar, Matthias Briel, Christiane Pauli-Magnus

**Affiliations:** 1 Clinical Trial Unit, Department of Clinical Research, University Hospital Basel, Basel, Switzerland; 2 Department of Medicine, Biomedicine and Clinical Research, Neurology, University Hospital Basel, Basel, Switzerland; 3 Novartis Pharma, Basel, Switzerland; 4 Institute for Biomedical Ethics, University of Basel, Basel, Switzerland; 5 Basel Institute for Clinical Epidemiology and Biostatistics, Department of Clinical Research, University Hospital Basel, Basel, Switzerland; 6 Department of Clinical Epidemiology and Biostatistics, McMaster University, Hamilton, Canada

**Keywords:** Monitoring, Risk-based, Risk proportionate, On-site monitoring, Quality Assurance, Academia

## Abstract

**Background:**

In spite of efforts to employ risk-based strategies to increase monitoring efficiency in the academic setting, empirical evidence on their effectiveness remains sparse. This mixed-methods study aimed to evaluate the risk-based on-site monitoring approach currently followed at our academic institution.

**Methods:**

We selected all studies monitored by the Clinical Trial Unit (CTU) according to Risk ADApted MONitoring (ADAMON) at the University Hospital Basel, Switzerland, between 01.01.2012 and 31.12.2014. We extracted study characteristics and monitoring information from the CTU Enterprise Resource Management system and from monitoring reports of all selected studies. We summarized the data descriptively. Additionally, we conducted semi-structured interviews with the three current CTU monitors.

**Results:**

During the observation period, a total of 214 monitoring visits were conducted in 43 studies resulting in 2961 documented monitoring findings. Our risk-based approach predominantly identified administrative (46.2%) and patient right findings (49.1%). We identified observational study design, high ADAMON risk category, industry sponsorship, the presence of an electronic database, experienced site staff, and inclusion of vulnerable study population to be factors associated with lower numbers of findings. The monitors understand the positive aspects of a risk-based approach but fear missing systematic errors due to the low frequency of visits.

**Conclusions:**

We show that the factors mostly increasing the risk for on-site monitoring findings are underrepresented in the current risk analysis scheme. Our risk-based on-site approach should further be complemented by centralized data checks, allowing monitors to transform their role towards partners for overall trial quality, and success.

**Electronic supplementary material:**

The online version of this article (doi:10.1186/s12874-017-0308-6) contains supplementary material, which is available to authorized users.

## Background

Adherence to the International Conference on Harmonization of Good Clinical Practice (ICH GCP) guidelines should ensure the safety, rights, and integrity of trial participants as well as the confidentiality of personal information and data quality [1]. Trial monitoring through trained clinical monitors is requested by ICH GCP, but the guideline provides limited insight on the procedures of quality assessment during such monitoring visits [2, 3]. Traditional approaches relied on intensive on-site visits and 100% source data verification (SDV) irrespective of the risk levels in the study, which have been associated with high cost and limited contribution to clinical trial data quality [4–6].

Recent developments at international bodies and regulatory agencies such as the European Medicines Agency (EMA) have supported the need for risk-proportionate approaches to clinical trial monitoring [7–9]. In November 2016, the ICH published the final version of the integrated addendum to ICH-GCP, advising Sponsors to develop a systematic, prioritized, risk-based approach to monitoring clinical trials [3]. Similarly, the forthcoming European Union (EU) Clinical Trial Regulation will permit reduced monitoring for low-risk intervention trials [10]. Among the first, the Risk ADApted MONitoring (ADAMON) Project proposed an instrument for the facilitation of risk analysis allowing on-site monitoring strategy tailored to the risk profile of every trial [11]. Risk analysis thereby refers to the risk of jeopardizing patient safety and rights or the validity of results and considers patient, site, and study design robustness-related indicators. Furthermore, risk analysis takes into account the risks of the study intervention compared to the risks a patient would run if treated in routine practice. This approach was first proposed in 2009 and later adapted by other stakeholders such as the Organization for Economic Co-operation and Development (OECD), the U.S. Food and Drug Agency (FDA), and EMA [9, 12, 13]. It encouraged study sponsors to assess, on a case-per-case basis, the risk associated with an individual trial protocol, implement risk assessments that focus on critical data and procedures, and utilize alternative monitoring approaches taking advantage of the increasing use of electronic systems. Sponsors should develop a monitoring plan that describes, based on the risk assessment, the monitoring strategy, the monitoring responsibilities of all the parties involved, the various monitoring methods to be used, and the rationale for their use [3]. However, in the absence of credible data to describe impact of a change of monitoring approach on data quality and study cost, the majority of industry-sponsored trials continue to be monitored using a traditional monitoring approach with up to 100% SDV. It has been estimated that SDV can consume up to 25% of the sponsor’s entire clinical trial budget, even though the association between data quality/subject safety and the extent of monitoring and SDV has not been clearly demonstrated [14]. Financial estimates of a single monitoring site visit range from US$800 in 1991 to US$1500 in 2009 [15, 16], with conservative cost estimates for one single query of US$150 [17]. The approach taken may therefore be evaluated as overcautious at best, and at worst, a complete waste of resources based on current reviews [18, 19].

In the academic setting, restricted resources often oblige investigators to apply a risk-based approach to trial monitoring which is expected to be less labor intense [20]. At the academic Clinical Trial Unit (CTU) at the University Hospital in Basel, Switzerland, we have applied risk-based on-site monitoring based on the ADAMON project for all patient-oriented research projects since 2012. In order to understand the implications of this approach for patient safety and data quality at our institution, we undertook this mixed-method investigation. The aim of our study was to i) retrospectively investigate the characteristics of findings documented during on-site visits, ii) identify key factors that might influence the number and types of monitoring findings, iii) assess the costs associated with our approach, and iv) understand the experience of our monitors and the challenges they face.

## Methods

### Setting

This mixed-method study was performed at the CTU of the University Hospital in Basel. The CTU offers monitoring services to investigator- and industry-initiated studies conducted at our institution and affiliated sites if desired by sponsors. CTU monitors are qualified by training and experience and work according to clearly defined standard operating procedures (SOPs) which are reviewed and updated by an autonomous quality-assurance officer on a regular basis. The risk evaluation adopted by the CTU (Table 1) includes a structured trial risk classification by the project manager according to the ADAMON project and the Swiss Human Research Act as described by the Swiss Clinical Trial Organization [21]. This approach allows the categories low, medium, or high risk; and the assessment of additional three risk modulators (Table 1). These risk modulators may lower or raise the risk within a certain risk category and therefore influence the duration of site visits, but not their frequency. After risk classification, the project manager specifies the extent and nature of on-site monitoring visits in the monitoring plan (Table 2). CTU monitors then conduct on-site monitoring visits according to the pre-specified monitoring plan and document monitoring findings in monitoring reports which are shared and discussed with both the sponsor and the project manager.

**Table 1:** Risk classification procedure at Clinical Trial Unit Basel

Risk classification procedure				Recommended systematic review of trial’s risk profile
1. Initial Risk classification	2. Categories of risk modulators	3. Risk classification	4. Modulators of monitoring extent	
Potential risk of therapeutic intervention in comparison to standard of medical care (as described in HRA & ADAMON): • Comparable (see also ClinO art. 19,20,61, category A) • Higher (see also ClinO art. 19,61, category B) • Markedly Higher (see also ClinO art. 19,20, category C)	Modulators from the following ADAMON categories may influence the initial risk category by a max. of +1 or −1 risk category (e.g. Intermediate to High Risk): 1. Potential trial participant-related critical indicators^a^ 2. Robustness related indicators – “hard primary endpoints” and/or simple clinical trial procedure 3. Site-related indicators^b^	An overall risk category is assigned based on the results of 1) and 2) as follows: • Low risk: Risk of intervention comparable & trial has at least one indicator of robustness and no participant related indicator • Intermediate risk: Risk of intervention comparable, or higher & trial has no indicator of robustness, or at least one participant-related indicator present • High risk: Risk of intervention higher or markedly higher, and trial has no indicator of robustness, or at least one participant related critical indicator present	The following modulators may influence monitoring extent (i.e. number of hours per visit) within risk category: 1. site experience with clinical trials 2. presence of an electronic database at site 3. whether site is coordinating lead site of the study	An additional risk assessment is required if the trial undergoes substantial amendments

1–3 conducted according to the Swiss Clinical Trial Organization Guidelines for Good Operational Practice V2.0. Scheme adapted from Hurley et al. [23]. ^a^Including indicators on vulnerability of study population, setting of recruitment, critical eligibility criteria, additional risks of therapy, trial procedures that are unusually complex, etc. ^b^Including essential technical, personnel, storage, transport, or documentation requirements at site. Site-related indicators do not affect the risk category of a study, but may modulate the extent and duration of individual monitoring visits. Human Research Act (HRA), ADApted MONitoring (ADAMON), Clinical Trials Ordinance (ClinO)

**Table 2:** Recommended on-site monitoring activities based on study risk classification. Informed Consent (IC)

Risk of Study	Initiation visit	Interim visit	Content of interim visits	Close out visit
Low	optional	after first patients, then adaptable (e.g. 1/year)	Endpoints (extent to be defined), IC (usually 100%)	optional
Intermediate	mandatory	after first patients, then adaptable (e.g. 1/year)	Endpoints (extent to be defined), IC (usually 100%), safety (usually 100%)	mandatory
High	mandatory	after first patients, then in regular intervals	Endpoints (extent to be defined), IC (usually 100%), safety (usually 100%)	mandatory

### Quantitative retrospective analysis

We included all investigator-initiated trials (IITs) and industry-sponsored studies monitored by the CTU between January 1st 2012 and December 31st 2014 with the exception of studies for which monitoring had never been fully initiated (i.e. <10% of planned working hours completed because of an early study discontinuation or delayed study start). Since the introduction of risk-based monitoring at our institution in 2012, a total of six monitors had been involved in monitoring activities. For all included studies, we extracted a set of variables covering detailed characteristics at the level of the study itself, the level of the study site and the individual monitoring visit.

Study-specific variables included

- study design,
- study type,
- study sponsor,
- type of research,
- study phase (I-IV), and
- type of study population (e.g. inclusion of vulnerable populations).

Variables covering study site information included

- site location,
- ADAMON risk category,
- presence of electronic database,
- principal investigator, and whether he/she changed during conduct,
- staff experience, and
- number of planned patients at the site.

At the level of the individual monitoring visit we extracted information on

- type of visit (i.e. initiation, interim, close-out),
- the number of
- administrative,
- patient rights,
- patient safety,
- laboratory/biological specimen,
- data point confirmation, and
- endpoint related findings.

Extraction and categorization of findings was performed independently and in duplicate (AO, MV, CB) from monitoring plans and reports using a validated web-based database (secuTrial®). Classification of findings corresponded to the main categories used in our monitoring reports and categories were treated as mutually exclusive. Table 6 provides examples of findings for each category. Discrepancies between extractors in classifying the variables were resolved through discussion by the extractors. After an initial calibration phase, agreement between the extractors was considered “good” if no more than four out of 49 extracted variables differed. Findings that were corrected immediately on-site were often not documented and therefore not included in our study. We summarized the number of findings descriptively, stratified by key variables and graphically displayed as i) total findings per study (or site, depending on the variable), ii) percentage of administrative or patient right findings out of total number of findings. Figures were interpreted visually. Furthermore, we collected information on human and financial resources employed for monitoring activities from the CTU Enterprise Resource Management (ERP) system for each project. We calculated total resource use by summing the total hours worked by our monitors during the analyzed time period (2012–2014), as retrieved from the ERP, multiplied by the hourly salary rate. We then divided the total human resource cost by total number of findings which we had documented and extracted from monitoring reports.

### Semi-structured interviews

We interviewed three monitors who were involved in these monitoring visits and who continue to work at our institution at present. The main themes covered during these interviews were i) monitors’ perspective on risk-based monitoring per se, ii) the practical settings in which these visits and findings of events were documented, iii) the challenges they faced during these visits, and iv) their perspectives on the future development of risk-based monitoring. As interviews did not include health-related data and were therefore not within the scope of the applicable Human Research Act (HRA, Art.1), we did not require formal ethical approval. Each interview was conducted in German by NR, tape recorded with the monitor’s permission, transcribed in full, and anonymized at the level of transcription. We examined all the transcripts in duplicate (BvN and NR) and BvN coded each interview. We then grouped codes into clusters around similar and interrelated themes until we reached consensus. In the results section below, we will describe and discuss three key themes that emerged from our qualitative interviews (a) factors influencing risk-based monitoring findings; (b) the monitoring process and the challenges faced; and (c) the current role of monitors and future perspectives.

## Results

### Study sample characteristics

We included forty-three studies (39 investigator-initiated, three industry-sponsored) monitored between January 1st 2012 and December 31st 2014 for analysis. Characteristics of these studies are shown in Table 3, study stratification by risk categories and associated risk factors in Table 4.

**Table 3:** Study sample characteristics (number, %)

		Total	
		n	%
Total studies		43	100
Study design	Interventional	34	79.1
	Observational	9	20.9
Study type	Multicenter	10	23.3
	Singlecenter	33	76.7
Study sponsor	Investigator (academic)	40	93.0
	Industry	3	7.0
Type of research	Drug	29	67.4
	Medical Device	5	11.6
	Biological Samples^a^	4	9.4
	Other^b^	5	11.6
Study phase (drug studies, *n* = 29)	I	9	31.1
	II	7	24.1
	III	8	27.6
	IV	3	10.3
	Other^c^	2	6.9

Study sample including 43 studies monitored by the CTU Basel between 2012 and 2014

^a^Biological samples incl. physiological or genetic analysis of human biological samples (e.g. urine, blood, tissue, etc.)

^b^Other incl. observational research, health economics assessments, or tissue-based intervention/stem-cell transplantation

^c^Other incl. cost-effectiveness trials not specific to a phase

**Table 4:** Study sample by risk categories and associated risk factors

		Total	
		n	%
Total studies		43	100
ADAMON risk category	Low	11	25.6
	medium	23	53.5
	High	9	20.9
	Total	43	100
Electronic database present at first patient in	Yes	19	44.2
	No	24	55.8
	Total	43	100
Principal Investigator change during study	Yes	3	7.0
	No	40	93.0
	Total	43	100
Vulnerable study population^a^	Yes	7	16.3
	No	36	83.7
	Total	43	100
Total sites		94	100
Staff experienced^b^, by site	Yes	88	93.6
	No	6	6.4
	Total	94	100
Staff change, by site	Yes	11	11.7
	No	48	51.1
	Unknown	35	37.2
	Total	94	100

Study sample including 43 studies monitored by the CTU Basel between 2012 and 2014, stratified by ADAMON risk categories, and factors associated with risk evaluation

^a^Defined as “children, adolescents, adults lacking capacity in the consent procedure, pregnant women and in-vitro fertilized embryos and fetuses, prisoners, and subjects in emergency situations” (according to HRA, Chapter 3)

^b^Defined as a) GCP trained, b) solely dedicated to research activities (e.g. a study nurse, resident, etc.), and c) has been involved in the conduct of one or more clinical research studies before

### Characteristics of monitoring findings

In total, we documented 2961 findings during 214 monitoring visits in 43 studies between 2012 and 2014 (Tables 5 and 6). In ten out of 43 studies, we monitored more than one site. Overall, administrative findings (46.2%; e.g. missing CVs or incomplete Investigator Site Files etc.) were equally predominant as patient rights findings (49.1%; e.g. wrongly signed and dated informed consent forms), whilst patient safety issues were found only exceptionally (1.1%). Although the studies varied in their total amount of findings, we documented at least one administrative and one patient right finding in almost every study, and at least one safety finding in a fifth of all studies (Tables 5 and 6). The remaining findings included issues related to laboratory procedures or biological specimen (2.3%), and issues related to the endpoint which could not be clarified with staff at site and required written confirmation (e.g. clarification of a questionable laboratory value which seemed out of range, 1.2%) (Table 5).

**Table 5:** Characteristics of monitoring findings

		Total	
		n	%
Total Studies		43	100
Total Monitoring Visits		214	100
Findings			
	Administrative	1367	46.2
	Patient rights	1453	49.1
	Patient safety	32	1.1
	Laboratory/biol. specimen	70	2.3
	Endpoint related data point: confirmation requested	36	1.2
	Endpoint related data point: Data point changed	3	0.1
	Total	2961	100
Average n findings/visit		13.8	
Studies with			
	at least 1 administrative finding	43	100
	at least 1 patient right finding	41	95.3
	at least 1 patient safety finding	9	20.9

Sum of findings by CTU monitors in total (number, %). *Note:* Findings which were resolved on-site between monitor and study staff and not documented in monitoring reports are not listed.

**Table 6:** Examples of monitoring findings

Finding category	Examples of findings
Administrative	• Changes at the investigational site (staff training, staff CVs, address, technical equipment, etc.) not documented • Functions and responsibilities log not up to date • Subject related logs not up to date • CRFs not available at site and/or not documented by authorized staff
Patient rights	• Informed Consent Forms not signed and/or not dated correctly • No valid and approved version of Informed Consent Form used • Amendments/addenda to Informed Consent Form not communicated to patients and no re-consent obtained • Patient did not fulfill all inclusion criteria
Patient safety	• No description of the process for detecting and reporting serious and unexpected adverse events and/or unanticipated problems involving risk to participants in place at site • Adverse events not correctly documented and/or reported as required (e.g. to Sponsor, EC, Competent Authority) • New safety information not approved by authorities • Staff not trained according to new safety information
Laboratory/Biological Specimen	• Biological specimen not stored correctly according to protocol • Process conducted not in accordance with Good Manufacturing Practice (GMP)
Data point confirmation requested	• Indicates whether finding challenges the credibility of data point, e.g. by stating “Please confirm that blood pressure measure is 100/65 mmHg”
Data point changed	• Indicates whether data point was adjusted as direct consequence of finding, e.g. “Blood pressure of 100/65 mmHg was corrected to 120/80 mmHg”

*CV* Curriculum Vitae, *CRF* Case Report Form, *EC* Ethics Committee

### Influencing factors on number and type of findings

**Fig. 1:**
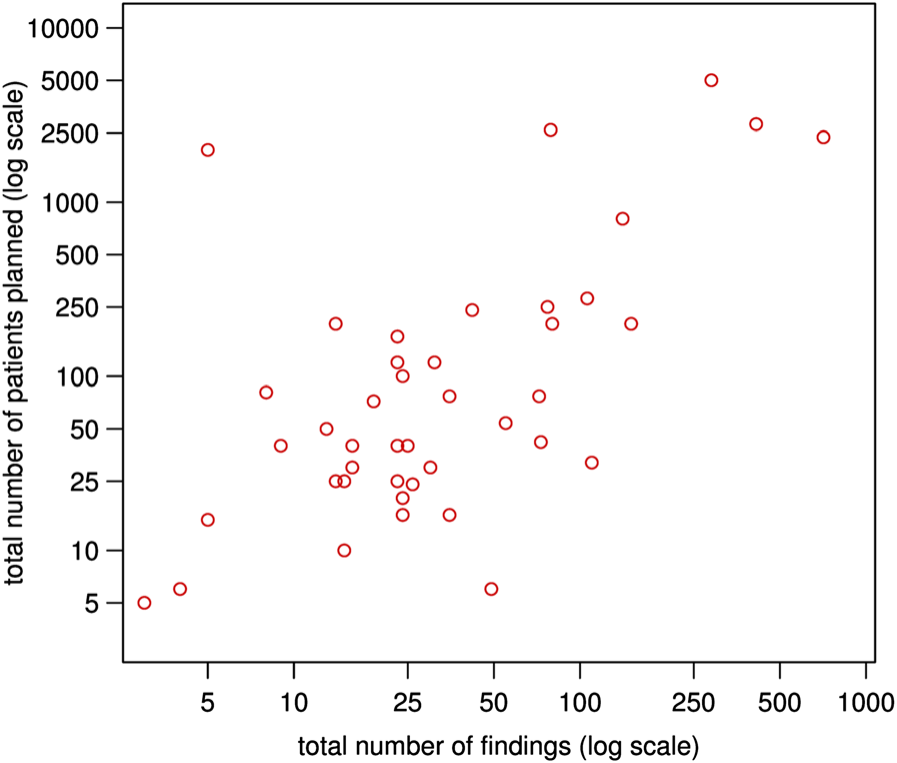
Studies according to the planned sample size and the final total number of monitoring findings (log scale)

**Fig. 2:**
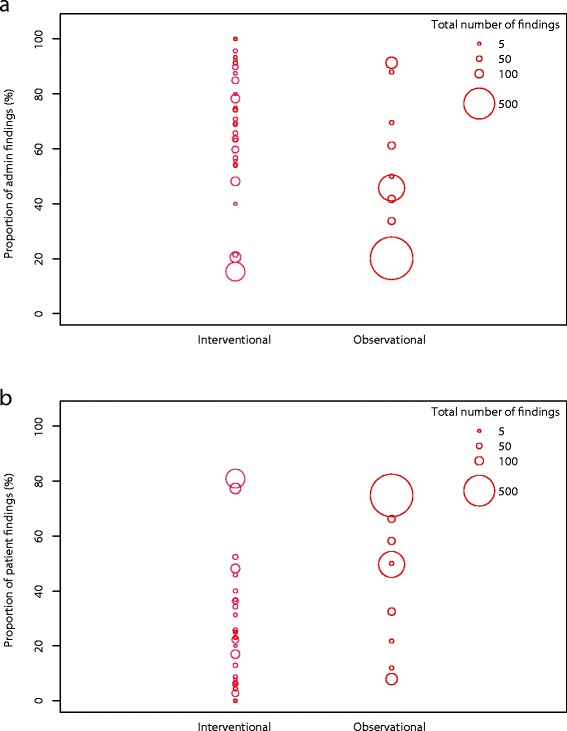
Total number of findings and proportion of administrative **a** and patient rights **b** findings in interventional and observational studies, by study. Diameter of circles proportionate to total number of findings per study

**Fig. 3:**
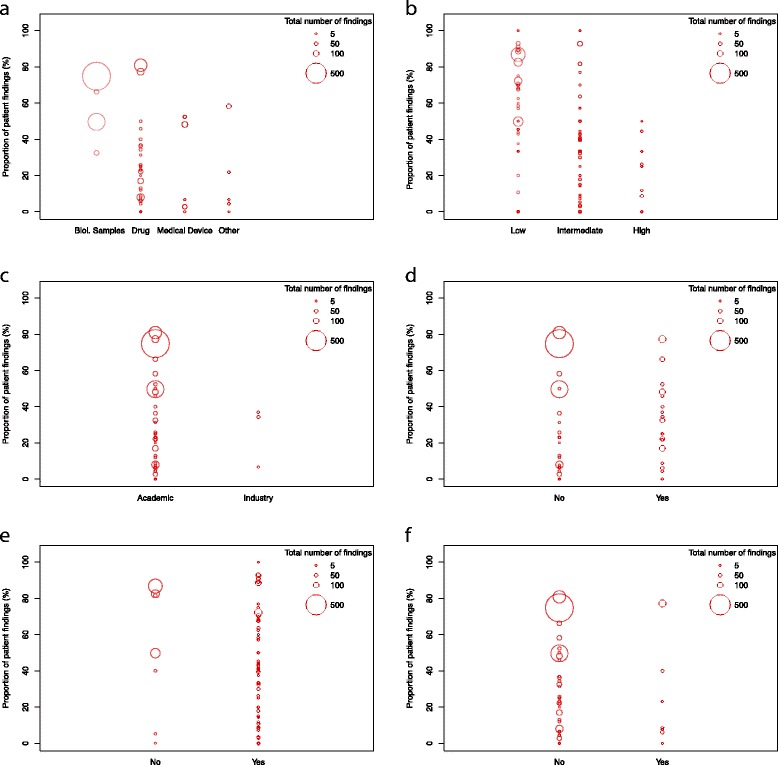
Total number of findings and proportion of patient rights findings in studies stratified by **a** study type, **b** ADAMON risk category, **c** study sponsor, **d** studies with vs. without electronic database, **e** studies conducted at sites with vs. without clinical research experience, **f** studies with vs. without vulnerable study population. Diameter of circles proportionate to total number of findings per study (**a**, **c**, **d**, **f**) or per site (**b**, **e**)

Generally, the sample size of a study was positively associated with the total number of findings (Fig. 1). Due to the low number of other than administrative and patient rights findings, the figures display both the percentage of patient rights (X %) and administrative findings (100–X %). Visual inspection of figures showed that factors such as observational study design (Fig. 2a, b), high ADAMON risk category (Fig. 3b), industry sponsorship (Fig. 3c), the presence of an electronic database (Fig. 3d), experienced site staff (Fig. 3e), and inclusion of vulnerable study population (Fig. 3f) were associated with lower numbers of monitoring findings. As a trend, studies sponsored by industry or with a high risk category tended to result in less patient rights findings compared to other studies, for which the proportion pattern (patient rights vs. administrative) varied widely (Figs. 3b, c).

Although observational studies generally resulted in fewer findings, two of the nine analyzed studies were outliers (>400 findings/study) (Fig. 2a, b). One was a multicenter study including seven sites but no electronic data capture system, resulting in a total of 413 findings (45.8% administrative, 49.6% patient rights) in seven initiation, seven interim one, and five interim two visits. The second study was a large single center study (>2,000 planned patients) with inexperienced study personnel and principal investigator. No initiation visit was performed given the low risk character of the study and an electronic data capture system was not available. In this study, four interim visits resulted in a total of 710 findings of which 20.1% were administrative and 75% were related to patient rights issues (Fig. 2b, largest red circle). In addition, both studies experienced a change in monitor, after which the overall number of findings increased.

Out of 43 monitored studies, 39 were monitored more than once (at least one site), and 12 were monitored at least three times (at least one site) (Additional file 1: Figure S1a). Thereof, 11 studies had an initiation visit and four studies experienced a change in monitor throughout the study. Generally, findings tended to decrease after the second interim visit. One study increased in findings after the third visit which was due to a new version of the informed consent which was not adequately used in all patients. The proportion of administrative findings was high at initiation but showed a decrease during the conduct of the study, whereas patient rights findings increased. (Additional file 1: Figure S1b, c).

In our sample, only three sites conducted three or more different studies within the given two-year time period (Additional file 1: Figure S2; site 1: studies 3, 4, 8, and 9; site 2: studies 22, 23, 33, and 36; site 3: studies 26, 21, and 32). Factors such as study design (interventional vs. observational), study type (e.g. phase 1–3), sample size, the risk associated with the studies performed (and therefore the associated monitoring risk category), and staff experience does usually not differ much within a given site, and visual inspection did not reveal a major difference in total number of findings within trials at each of the three sites (Additional file 1: Figure S2). However, the mentioned characteristics differed significantly across the three sites (one high risk pharmacological phase 1 unit, one low risk observational cardiology unit, and one medium risk cognitive neuroscience unit) and did therefore not allow for comparison between these sites. In the ten multicenter studies included in our sample (1, 7, 12, 14, 16, 19, 21, 26, 31, and 32 in Additional file 1: Figure S2), no trend in total number of findings across sites could be identified.

### Cost of monitoring

Data on monitoring costs were only available since May 2012, when an electronic enterprise resource management system was implemented at the CTU. Overall, cost data was available for 33 out of 43 monitored projects. For these projects, we documented a total of 4320 working hours for 2401 monitoring findings. With an hourly salary rate of US$92.5 this translated into total personnel costs of US$ 399’280 and an average per findings cost of US$166. In this estimate, however, the endpoint related findings that were resolved on-site are not considered, leading to potential overestimation of costs per finding.

### The Monitor’s perspective

In addition to evaluating the characteristics of findings of our on-site visits, we aimed to understand the practical experience of monitors involved, challenges they face during monitoring, their perspectives on the risk-based approach, and suggestions for improvement.

Three monitors we interviewed had been working as clinical trial monitors for two to 21 years, two of them mainly in the academic setting whereas the third one mainly in the pharmaceutical industry environment. With the introduction of risk-based monitoring at the CTU in 2012, all three participants started to monitor according to the above described standard procedure. Below we describe three main themes from these interviews and provide select quotations for each.

### Factors influencing risk-based monitoring findings

All three monitors expressed a generally positive attitude towards the concept of upfront risk evaluation, which allows assessment of critical factors in the study design or practical challenges that the study team might face while implementing the trial as described by one of our monitors.

*“The positive effect clearly is that you think more about the study itself. If you take the effort to classify the study by risk factors you can actually eliminate many things upfront. Because of that evaluation, I know what to set value on when I open a site”. (Monitor III)*

The factors that the monitors in general deemed crucial for low numbers of findings in trials were professional, trained and motivated study personnel, together with a robust study design and rigorous planning of a study. These factors were also attributed to support participant recruitment into the trials and eventual success of the trial. Monitor III argued that indicators related to the study site were underrepresented in the ADAMON risk evaluation, in spite of the fact that they have significant influence on the way trials are conducted. Other factors that the monitors believed to contribute positively to overall trial quality and success were the quality of the study protocol, the early involvement of monitor and study nurses in protocol development, training and experience of all personnel involved, planning of finances and infrastructure before trial begins, available resources, trial coordination and management, assignment of clear responsibilities, well planned recruitment, and clear and transparent communication among all stakeholders involved as elaborated in the quote below.

*“But it all depends on the experience of staff on-site, if they have lots of experience with studies, they know how to do it. But don’t forget that staff changes so often at the site, you never get the same people from the start until the end of a study. The new ones, how will they be trained? We as monitors only hear about it half a year later and if you only visit them once a year, you hear that they have changed the recruiting physician and that patients have been informed wrongly for three quarters of a year.“(Monitor III)*

### Monitoring process and challenges faced

With respect to what the current risk-based approach is able to cover on-site, all monitors came up with two distinct topics, namely patient safety and rights, and data quality. Monitor I and II felt that minimum aspects of patient safety and rights, incl. informed consent forms and inclusion/exclusion criteria, but also “crucial” data points such as the primary endpoint, were mostly covered by their on-site visits. Two monitors did not see any issues related to patient safety or their rights with the current approach as described below.

*“It depends on the monitoring plan; usually we look at 100% of the Informed Consent Forms, unless there are too many patients such as in cohort studies. We always look at the inclusion and exclusions criteria. Endpoints are to be discussed and defined with the principal investigator. Depending on the budget available, we might also look at the Trial Master File, and then I am done in no time” (Monitor I)*

*“I hope that I cover safety aspects with my monitoring. Actually I don’t see any issues with it. There is no monitoring plan without the safety aspect, usually it is 100% covered. (…) Depending on what you find, you adapt it (the monitoring plan). If you find critical issues, for example a Serious Adverse Event that was not documented, you tell the team (on-site) to look at the other patients’ data and check whether they were correct.” (Monitor II)*

However, Monitor III expressed concerns about rather infrequent on-site visits with long time gaps in between during which there was a clear risk of missing patient safety or patient rights aspects in particular. He also feared that these findings would then get resolved only during the next visit which might be after six months.

*“I question whether this approach is compatible with GCP. According to GCP you put patients first, and then the scientific question. There you also include data protection and the ICF (informed consent form). You don’t really respect patient rights if, for example, a wrong ICF version was signed and I only notice after half a year, just because the study is a low risk study according to the evaluation. I don’t think it affects the scientific validity, but more the patient safety and rights aspects”. (Monitor III)*

With respect to data quality, all monitors mentioned “systematic data errors”, i.e. errors that are not produced by chance. They were concerned to miss systematic errors with the risk-based approach. Monitor I sometimes preferred to monitor more frequently in order to identify systematic data errors as and when they occurred. Monitor I and II would like to rather cover 100% source data for fewer patients than single puzzle pieces of several patients to be able to pick up systematic errors as described in quote below.

*“One is for sure, if you don’t see that mistake at the beginning, it’s going to repeat with the next patients. So that’s a systematic error then, and of course data quality suffers! “(…) “If I knew that every three month there is a monitor at your doorstep who wants to critically look at your data, then I am of course required to get my stuff done in time and…a bit more accurate as if, you know, I know that there is anyways no one looking at my data. Then you get the running around after the data at the end of the year”. (Monitor III)*

Monitor II further questioned low importance given to “less important data points” (e.g. lab values not specified as outcome variables). These “less important data points” are not considered in the risk-based monitoring plan according to ADAMON and therefore not checked by the monitors

*“Often, we do not look at lab values because they are not seen as risky values, maybe only 10% is seen as crucial for the primary endpoint. But then you ask yourself whether you wouldn’t miss transcription errors if you don’t look at these values at all. (…) There are not only systematic errors that you don’t see but also those that appear everywhere and they’re even more difficult to detect. And it also depends on the format in which you collect data, if it is on paper or not. The CRF (case report form) heavily influences the number of mistakes that are made, and you don’t look at all of these with the risk-based approach. (…) If you, for example, look at one patient 100% in a study and not the others, you of course detect systematic errors and point to that and make them look at the other patients in their documentation as well.” (Monitor II)*

All monitors believed that data quality would improve with increased frequency of monitoring. Monitor I and III disagreed with the current guidelines that in some low-risk cases, an initiation visit is not necessary. They would rather leave out the close-out visit but always perform an initiation visit to train the personnel on crucial GCP and study-related aspects.

*“I assume that in an inspection, you know, if they were to look at 100% of the data after my monitoring, they would find errors even in my monitored data. But I am sure you see exactly what documents were monitored and which weren’t. (…) but I believe that data quality would for sure be better with more monitoring, there I am 100% sure.” (Monitor I)*

*“Also the change in personnel has an influence, if you don’t take care of the training of new employees, and you don’t involve them. The situation on-site is not reflected adequately in ADAMON, it’s more sort of a weak factor, that if you’re there anyways, you monitor longer, but it doesn’t influence the frequency of monitoring. Changes and structures on-site should have a stronger influence on the frequency of visits, in my opinion. “(Monitor III)*

Monitor III further questioned whether in reality the risk-based approach proves cost-effective if many errors were missed in between visits due to the low frequency of visits and when amendments are needed to correct those.

*“It is not clear whether it is cheaper if the monitor visits less frequently and everything on-site goes downhill or if it wasn’t better if the monitor had visited once or twice more, you know. To make crappy data better again is also not cheap, right? Even if you adapt all ADAMON criteria, we should get away from only seeing cost savings in it. “(Monitor III)*

When monitors were further asked about why the frequency of monitoring visits could not be adapted in cases where needed, all mentioned the difficult financial environment in which academic trials are conducted. In their experience, most often, funding limitations were the main factor for restrictions in the amount and frequency of monitoring visits that could be performed, rather than the actual risk categorization.

*“…just because the budget doesn’t allow, you know, I would like to monitor more frequently or follow the risk classification more strictly, but you can’t, because of the financial limitations.” (Monitor I)*

#### Role of monitors and future perspectives

We explored further monitors’ views on ways to improve monitoring process and to make it cost effective. All monitors discussed few possibilities especially in defining their role as monitors and the way they are perceived by the study teams. They would like to be seen as trustworthy partners who assist in ensuring trial success and quality instead of being mere “controllers”. They hoped that principal investigators would be more familiar with their role and study teams will not see them as a “necessary evil” who consume significant part of the study budget and with whom they have to deal with, but rather as supporting partners who assist in achieving the study goals and ensure study quality. They see themselves as critical examiners, a fresh pair of eyes, who provide constructive feedback to the study team, as trusted supporters, facilitators of communication across sites, and motivators.

*“I would wish for more acceptance of monitoring, they all think it is just a necessary evil. That you don’t do it because it’s important and helps data quality, but only because the regulators and authorities want it from you. I am sure, often, monitoring reports are not even read. There should be more trust that we help data quality and therefore, also help the answer to the study question”. (Monitor II)*

*“I always tried to make them feel like I am their partner, and not some teacher or something…I don’t want to point the finger at them, but build a trustworthy relationship so that people at the site know they can trust me. So that they know they can call me if there is a problem. (…) The aim is to make them understand that we are partners and try to help to get to a good result that all of us do a good job, and that patients are safe and their rights are protected”. (Monitor III)*

## Discussion

As far as we are aware, this is the first mixed methods study to retrospectively investigate the outcome of on-site risk-based monitoring according to the ADAMON framework with regards to patient rights, safety and data quality in a sample of 43 interventional and observational studies in the academic setting. We identified a proportionate amount of patient rights and administrative findings, while findings concerning patient safety were rare, resulting in costs per documented finding of US$166. While administrative findings naturally predominantly occurred at the beginning of the studies (e.g. at initiation visit), patient rights findings developed proportionately with the proceeding enrolment of patients in the study.

Based on our study sample we found in exploratory investigation factors such as observational study design, industry sponsorship, a high risk classification, and the inclusion of a vulnerable study population to be associated with fewer findings during on-site visits. Surprisingly, a high risk category per se and the inclusion of vulnerable study populations, which come with an increased frequency of on-site monitoring visits according to ADAMON, do not cause a larger number of total findings. Counterintuitively, high risk studies therefore seem to be at lower risk for poor quality, probably due to the more closely monitored regulatory and legal environment which supports a well-planned set up of the study. It is therefore questionable whether the current monitoring scheme according to pre-set risk factors will be effective, both for quality and cost of trials, unless we learn from these findings. An alternative approach could, for example, be “experience-based” in the sense that monitoring frequency and extent are continuously adapted after on-site visits depending on the findings that have occurred. This would certainly allow for more flexible monitoring strategies when and where on-site visits are actually needed in line with current “quality-by-design” initiatives [22–24]. Practically however, changes in contracts are often difficult after monitoring plans have been written and budgets have been allocated.

Further, our results are in line with a systematic review of risk-based monitoring tools which states that both ADAMON as well as the SCTO guideline do not assess all 12 fundamental risk indicators as described in the recently published risk indicator taxonomy for supervision of clinical trials on medicinal products [25, 26]. While ADAMON lacks indicators on professionalism, reputation, and level of experience of investigator, clinical trial site, and sponsor, the SCTO guideline provides indicators assessing the level of experience at least to some extent [26]. We show that the two of the three risk modulators that the CTU has used to adjust monitoring extent purely based on experience with previous studies, i.e. the absence of an electronic data capture system and the lack of experience of a site, are clearly associated with a higher number of findings. This was exemplified by the two observational outlier studies. These factors should therefore be considered not only in the modulation of the extent of monitoring, but also influence the frequency of on-sites visits.

Our qualitative enquiry highlighted that the involved monitors understand the positive aspects of a risk-based approach in the resource constrained academic setting, but fear to miss systematic errors or even patient right violations due to the low frequency of visits or the lack of a requirement for initiation visits in low risk studies. They stressed the importance of well trained, motivated and experienced trial personnel, i.e. the investigator, study nurses, and related site staff, for overall trial quality and success. They further exemplified that these human factors should play a larger role in the risk evaluation, and that ADAMON does not cover these aspects adequately. The additional factors that were mentioned to be crucial for trial quality and success predominantly covered the design of the study, including how well the practical aspects of the study are planned, and factors related to the functioning of the site, such as training and experience of personnel, planning of finances and infrastructure, resources, and recruitment, trial coordination and management, assignment of clear responsibilities and transparent communication. While some of these aspects covered by ADAMON, none of them has an influence on the final monitoring risk category. We therefore encourage to put more emphasis on site-related and personnel-related risk factors in the risk evaluation of any studies, both industry- and investigator initiated, in addition to any framework used.

The initial concept of risk-based monitoring aimed at optimizing the use of scarce resources while assuring patient rights and safety as well as data quality in accordance with the GCP guideline. Several years later, cornerstones of the risk-based monitoring concept, such as ADAMON and OPTIMON [8, 20], are still under evaluation and evidence on the effectiveness and cost savings of this approach in different host organizations, sponsors, settings, or trial designs remains relatively sparse [27, 28]. There is, however, emerging consensus that 100% SDV and dual entry procedures are time and cost inefficient in detecting data discrepancies [17, 29, 30], that some types of errors in a clinical trial are more important than others [6], and that a site monitoring approach tailored to the risk of the trial can be supported in order to detect critical issues [7, 8, 31]. With our approach, we mainly identified patient rights and administrative findings, for which we were not able to retrospectively judge how critical they were. Our monitors, however, clearly stated the fear of missing systematic data errors and even critical issues concerning patient rights during the on-site visits, which are restricted in frequency and extent by academic financial constraints, or a low risk category. We thus see a need for complementary quality assurance measures for systematic data errors that can be performed off-site, such as central data verification..

Recently, combinations of central data verification (e.g. patient rights and safety) and on-site monitoring strategies have been applied to improve the efficiency of risk-based procedures [5, 7, 32]. Compared to other tools, ADAMON does only provide vague guidance on the nature and extent of centralized monitoring, while the adapted form by the Swiss Clinical Trial Organization recommends that protocol compliance could be monitored centrally for low-risk trials, as described in a recent systematic review by Hurley et al. [26]. According to the FDA’s recommendations on risk-based monitoring for industry and the current integrated addendum to ICH-GCP, centralized monitoring processes could provide additional capabilities to on-site monitoring in the academic setting, thereby dispelling our monitor’s doubts on missing systematic data errors [3, 9]. Statistical monitoring methods are an area of active research and have been suggested to “help improve the effectiveness of on-site monitoring by prioritizing site visits and by guiding site visits with central statistical data checks” [32] and were shown to identify the great majority of on-site monitoring findings [7]. In line with our results, Tudur Smith et al. have recently described such an approach in non-commercial studies, allowing for “triggered”, rather than predominantly “routine” on-site visits [5]. However, empirical data on its effectiveness and the costs, advantages and disadvantages of alternative methods are still missing. With central monitoring strategies allowing for efficient data quality checks, monitors would then also be more flexible to transform their roles from “controllers” towards “partners”, as they had mentioned in the interviews, and contribute to overall clinical research quality rather than mere GCP-conformance.

We are aware of a number of limiting factors in our analysis as follows. First of all, the retrospective design of our study did not allow standardization of extracted data across studies and monitors. Our sample was heterogeneous in terms of study type, study design, intended sample size and the risk categorization. It may further not be entirely representative in type and size of all studies conducted at out institution, as we predominantly monitor investigator-initiated studies. However, we aimed to minimize selection bias by including all trials monitored by the CTU within a given time period. Second, due to the small sample size and large heterogeneity of studies, we did not perform multivariable analysis but summarized absolute numbers of findings descriptively. Third, these studies were monitored by six different monitors with varying degree of experience and over a period of two years. In spite of a standardized procedure, each monitor has his or her own personal monitoring style, different level of attention to detail and strive for perfection. These factors could have influenced their interactions with the study team, generation and documentation of monitoring related findings. Fourth, we could only interview three of these six monitors who continue to work at our institution. We are fully aware that their experience and perspective cannot be generalized but their perspective is critical in understanding the challenges in effective monitoring and ways to improve monitoring process. Furthermore, perspectives of additional stakeholders involved in risk-based monitoring (e.g. trial project leaders or principal investigators) should be considered in the future. Finally, we want to discuss the subjective and flexible nature of risk classification (e.g. risk compared to standard treatment, judging the experience of staff at a study site and adjustments to available budgets) and diversity in monitoring style of different monitors and their documentation practice. This could have contributed to an unknown number of findings that were resolved directly on-site without documentation and hence out of scope of current analysis. In addition, we were unable to calculate number of findings in relation to number of patients recruited due to inconsistent documentation of number of patients monitored during each monitoring visit.

However, by publishing our experiences, we are supporting an ancillary recommendation made by the Clinical Trials Transformation Initiative (CTTI) project on effective and efficient monitoring to “share knowledge and experiences, so that best practices may be established” [33]. The hurdles that we experienced in the generation of urgently needed evidence on effectiveness of monitoring strategies have provided food for thought as follows: Our study illustrates that while the very concept of the current risk-based approach allows for flexibility in tailoring monitoring to the requirements of a specific study, it may also be prone to ambiguity. The lack of a “one-fits-all” model may lead stakeholders to be at loss to define a relevant trial-specific monitoring strategy. We realized that the combination of flexibility in designing the monitoring approach and the subjectivity and individual preferences of monitors in documenting findings add to the complexity of analyzing the effectiveness of risk-based approaches. For instance, the change of monitor in a project was often associated with a specific findings pattern (e.g. an increase in findings), which may be explained by preferences regarding the individual documentation style, the level of detail monitored, or the monitor’s experience. This haziness including the inter-human and inter-institutional variability should receive more recognition in the field when investigating the effectiveness of risk-based approaches in the academic setting.

Finally, we perceive that all of the efforts invested so far focus on optimizing the cost-effectiveness of current strategies, i.e. assuring patient’s rights and safety as well as data quality in accordance with the requirements of ICH-GCP. Interestingly, none of the efforts has so far questioned the real impact of current monitoring activities in increasing the overall quality of academic clinical studies. In line with other authors we believe that the current detection of non-critical findings adds only little to overall study quality, while consuming significant resources that could be spent in areas known to be critical for successful trial conduct [17]. For instance, empirical data show that academic clinical research in particularly suffers from major hurdles in the recruitment of patients, with recruitment failure being the main reason for early discontinuation of trials [34–38]. According to a report by the Tufts Center for the Study of Drug Development, about 50% of sites fail to reach the planned recruitment targets and more than 95% of clinical trials do not end on time and on budget as planned in the first place. Ninety percent of the studies meet their recruitment goals, but at the expense of mostly twice as much time as originally planned [39]. Often, this is due to too many avoidable protocol amendments, with a first amendment implemented even before the very first patient has been enrolled [40]. Almost half of the protocol amendments are considered “somewhat” or “completely” avoidable [39]. The European Clinical Research Infrastructure Network (ECRIN) hence provides a broad definition of monitoring as activities which must be “understood as all onsite and central activities dealing with checks of data and procedures as well as with the overall surveillance and stimulation of the trial progress” [41]. The interviewed monitors have supported such a holistic approach starting with their involvement in protocol development and processes based on trial procedures rather than data points per se, supporting overall trial completion with conclusive results. In accordance with ICH-GCP E6 R(2), we envision the future monitor to be an on-site partner to the study team, supported by centralized data checks adaptable to the risk of a trial, considering the experience of and the management at the site itself [3].

## Conclusion

In conclusion, we show that the factors which mostly increased the risk for on-site monitoring findings are underrepresented in the current ADAMON scheme, but have partly been considered by our monitors based on their professional experience. We believe that the “human factor” has been underestimated in the evaluation of risk-based approaches so far, and should receive more recognition in the future. In line with recent developments, our risk-based on-site monitoring should be complemented by centralized data checks in the future, allowing monitors to transform their role towards partners for overall trial quality, and success. However, evidence on the methodology and the (cost-) effectiveness of different combinations of the two approaches is still sparse for the academic setting. Future research should therefore address urgently needed strategies for efficient and effective monitoring, based on the current knowledge on risk factors in the academic setting.

## Additional file

Additional file 1: Figure S1. Total number (a) and proportion of findings (b/c) over time, per site. In (a), only studies (sites) with 3 or more monitoring visits are presented. **Figure S2.** Total number of findings over time, by individual study and site. Circles depict monitoring visit, lines connect visits at one particular site. Circles that are not connected by lines depict monitoring visits at different sites. Number 1, 7, 12, 14, 16, 19, 21, 26, 31, and 32 are multicenter studies. If different sites are not distinguishable, the total number of findings at this particular visit was the same (superposed circles). (PDF 350 kb)

## Abbreviations

ADAMON: Adapted monitoring
ClinO: Clinical trials ordinance
CTTI: Clinical trials transformation initiative
CTU: Clinical trial unit
ECRIN: European clinical research infrastructure network
EMA: European medicines agency
ERP: Enterprise resource management
FDA: Food and drug administration
HRA: Human research act
IC: Informed consent
ICH-GCP: International conference on harmonization of good clinical practice
IIT: Investigator-initiated trial
SDV: Source data verification
SOP: Standard operating procedure
