# Supplementary figures and images for: Generating evidence on a risk-based monitoring approach in the academic setting – lessons learned
Source: BMC Med Res Methodol. 2017 Feb 14;17:26. doi: 10.1186/s12874-017-0308-6 (PMC5307807; doi:10.1186/s12874-017-0308-6)

1a

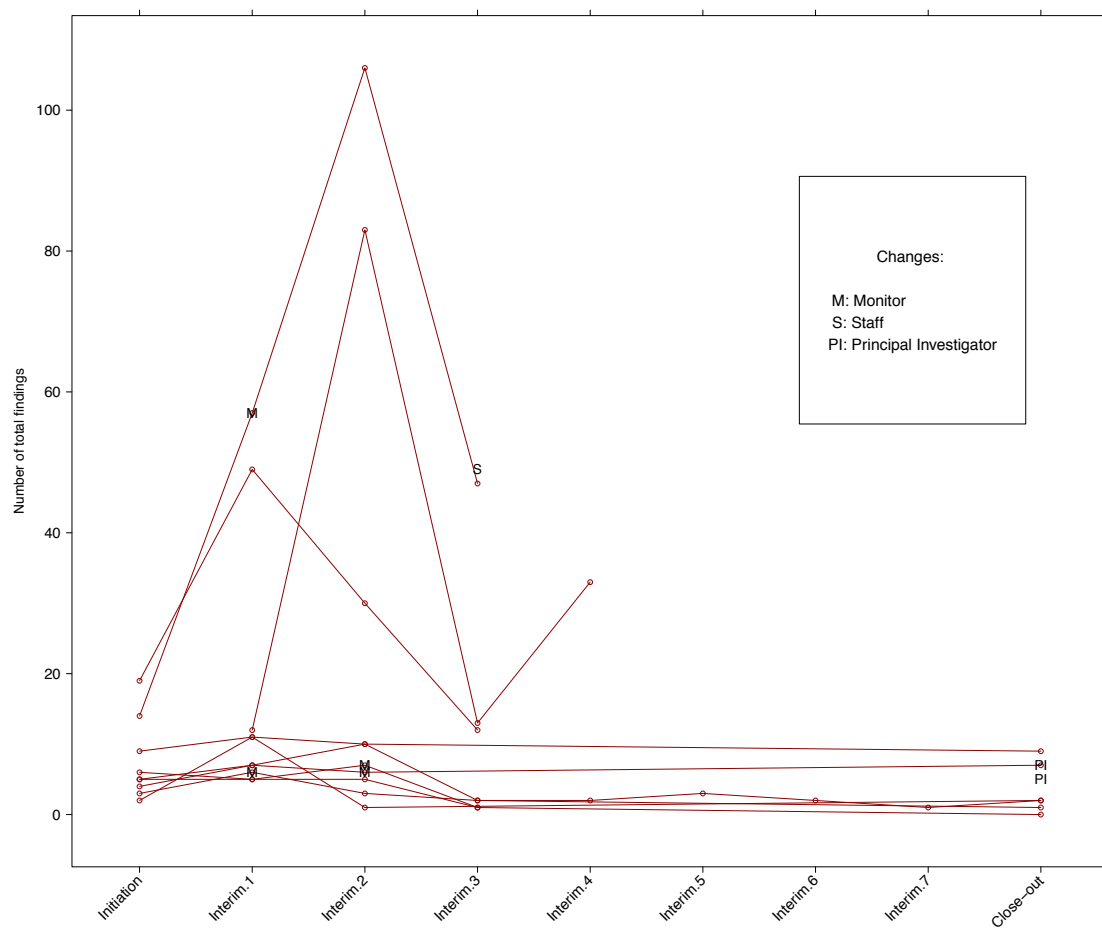

1b

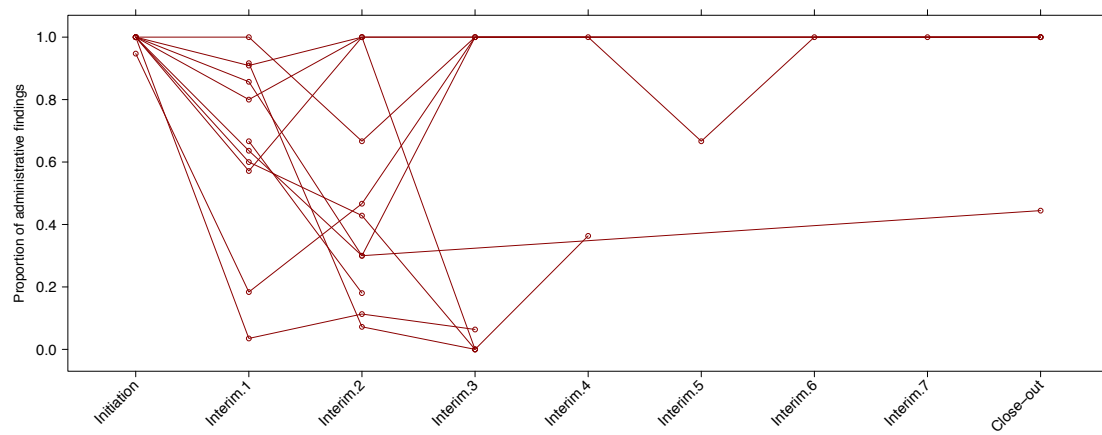

1c

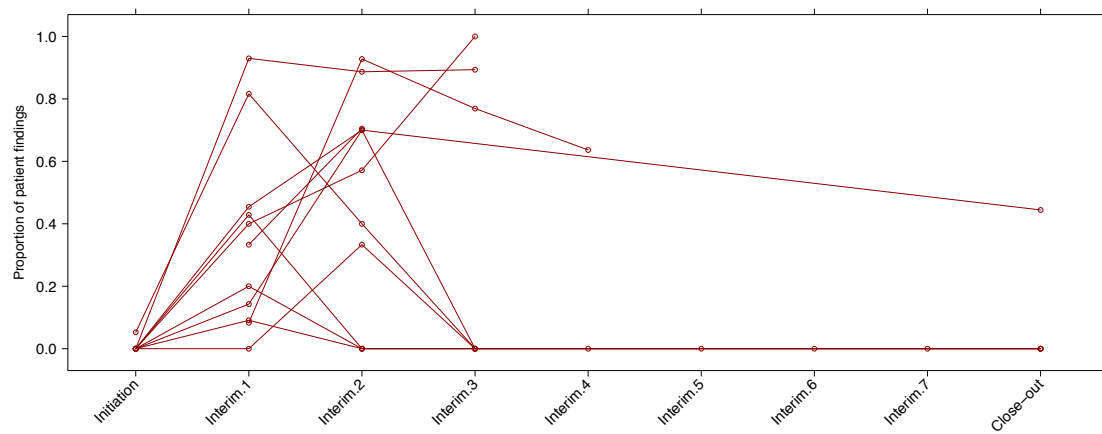

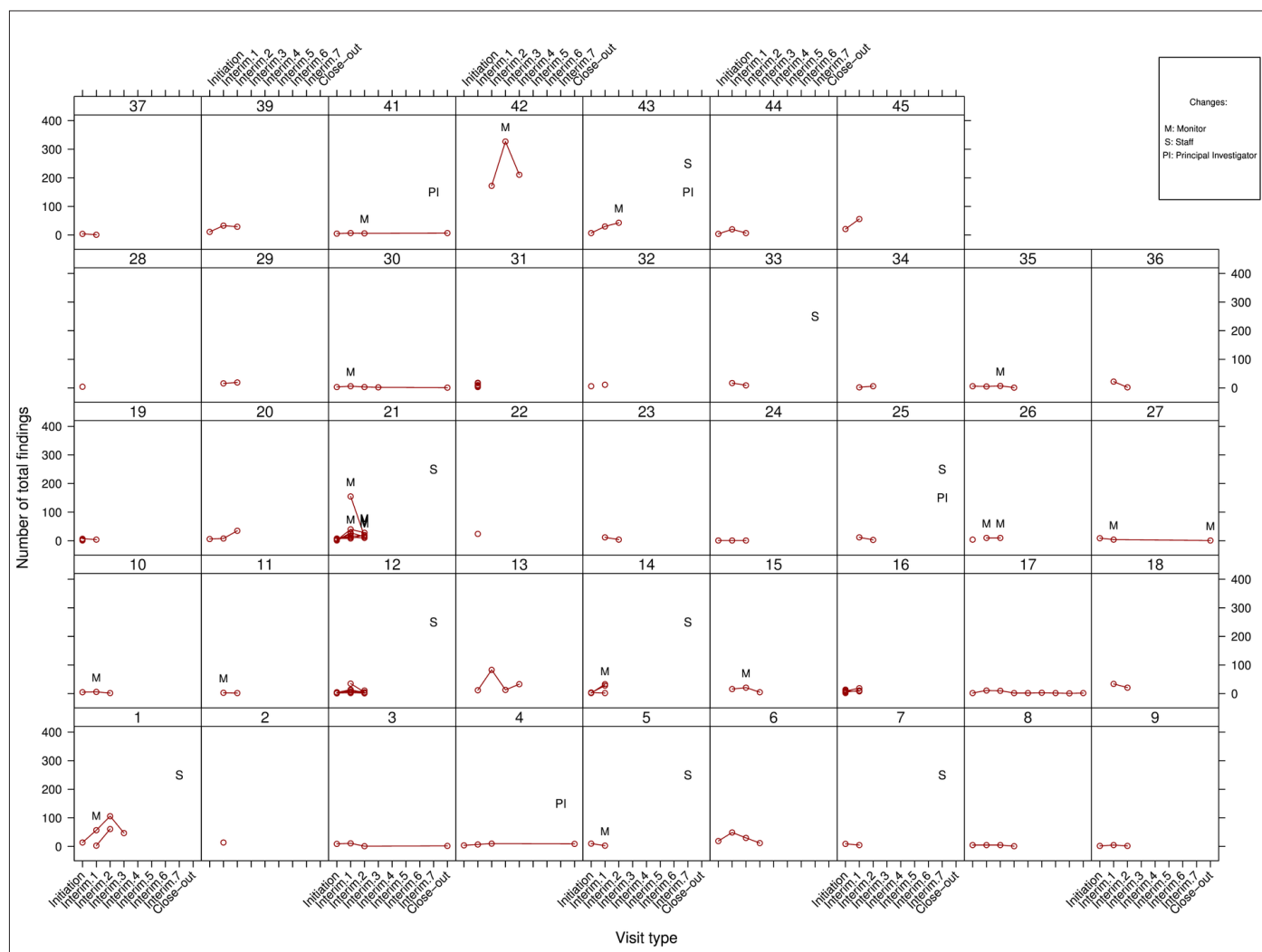

Supplement: Additional file 1: Figure S1. — Total number (a) and proportion of findings (b/c) over time, per site. In (a), only studies (sites) with 3 or more monitoring visits are presented. Figure S2. Total number of findings over time, by individual study and site. Circles depict monitoring visit, lines connect visits at one particular site. Circles that are not connected by lines depict monitoring visits at different sites. Number 1, 7, 12, 14, 16, 19, 21, 26, 31, and 32 are multicenter studies. If different sites are not distinguishable, the total number of findings at this particular visit was the same (superposed circles). (PDF 350 kb) [file 12874_2017_308_MOESM1_ESM.pdf]
